# Complete genome sequence of thermophilic *Bacillus smithii* type strain DSM 4216^T^

**DOI:** 10.1186/s40793-016-0172-8

**Published:** 2016-08-24

**Authors:** Elleke F. Bosma, Jasper J. Koehorst, Sacha A. F. T. van Hijum, Bernadet Renckens, Bastienne Vriesendorp, Antonius H. P. van de Weijer, Peter J. Schaap, Willem M. de Vos, John van der Oost, Richard van Kranenburg

**Affiliations:** 1 Laboratory of Microbiology, Wageningen University, Dreijenplein 10, 6703 HB Wageningen, The Netherlands; 2 Present address: The Novo Nordisk Foundation Center for Biosustainability, Technical University of Denmark, Kemitorvet, Kgs. Lyngby, 2800 Denmark; 3 Laboratory of Systems and Synthetic Biology, Wageningen University, Dreijenplein 10, 6703 HB Wageningen, The Netherlands; 4 CMBI, NCMLS, Geert-Grooteplein Zuid 26-28, 6525 GA Nijmegen, The Netherlands; 5 Corbion, Arkelsedijk 46, 4206 AC Gorinchem, The Netherlands

**Keywords:** *Bacillus smithii*, Genome sequence, Lactic acid, Thermophile, Thermophilic bacillus, Biotechnology

## Abstract

*Bacillus smithii* is a facultatively anaerobic, thermophilic bacterium able to use a variety of sugars that can be derived from lignocellulosic feedstocks. Being genetically accessible, it is a potential new host for biotechnological production of green chemicals from renewable resources. We determined the complete genomic sequence of the *B. smithii* type strain DSM 4216^T^, which consists of a 3,368,778 bp chromosome (GenBank accession number CP012024.1) and a 12,514 bp plasmid (GenBank accession number CP012025.1), together encoding 3880 genes. Genome annotation via RAST was complemented by a protein domain analysis. Some unique features of *B. smithii* central metabolism in comparison to related organisms included the lack of a standard acetate production pathway with no apparent pyruvate formate lyase, phosphotransacetylase, and acetate kinase genes, while acetate was the second fermentation product.

## Introduction

*Bacillus smithii* is a facultatively anaerobic, facultatively thermophilic Gram-positive bacterium, originally identified as *Bacillus coagulans* [1, 2]. Similar to its close relative *B. coagulans*, *B. smithii* has biotechnological potential, as it is able to ferment a range of carbon sources [2] into lactate and other green building block chemicals [3, 4]. The production of such green chemicals at elevated temperatures from lignocellulosic biomass has the potential to lower production costs of these chemicals. Compared to currently used mesophilic production hosts, such as Lactic Acid Bacteria or *Escherichia coli*, the amount of enzymes needed for hydrolysis of lignocellulose is ~3-fold lower around 50–60 °C, which is the temperature of moderately thermophilic temperatures [5]. Furthermore, fermentation at higher temperatures decreases contamination risks and cooling costs and increases product and substrate solubility [6, 7]. In order to enable the development of *B. smithii* as a platform organism, genetic tools were recently developed for it [3, 4]. To fully exploit the biotechnological potential of this species and to gain insight into its metabolic pathways, we sequenced the genome of the *B. smithii* type strain. Reconstruction of the central metabolic pathways based on the genome reveals some remarkable differences with its close relative *B. coagulans**.*

## Organism information

### Classification and features

**Fig. 1:**
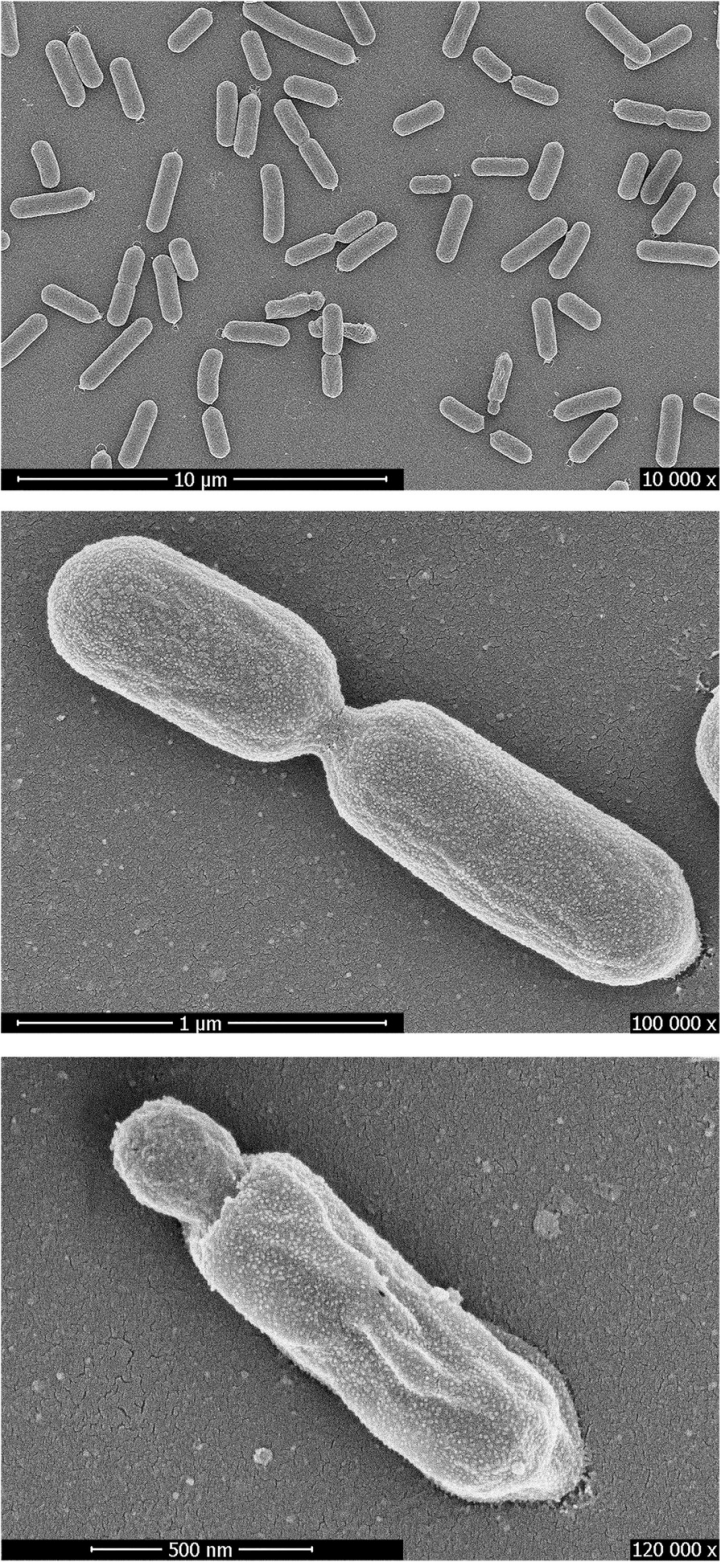
Scanning electron micrographs of *B. smithii* DSM 4216^T^

*B. smithii*DSM 4216^T^ is a motile, spore-forming, rod-shaped (0.8–1.0 by 5.0–6.0 μm [2]/0.5–1.0 by 2.0–6.0 μm, Fig. 1), facultatively anaerobic, facultatively thermophilic bacterium with wide ranges of both temperature (25–65 °C) and pH (5.5–7.0) [2]. An electron micrograph of *B. smithii*DSM 4216^T^ is shown in Fig. 1. Based on existing literature [2], HPLC analysis [3, 4] and API-tests, it is concluded that the species is able to ferment a range of carbon sources into mainly lactate, with acetate as the major by-product and minor amounts of succinate and malate (Table 1).

**Table 1:** Classification and general features of *B. smithii* DSM 4216^T^ according to MIGS standards

MIGS ID	Property	Term	Evidence code^a^
	Classification	Domain Bacteria	TAS [30]
		Phylum *Firmicutes*	TAS [31–33]
		Class *Bacilli*	TAS [34, 35]
		Order *Bacillales*	TAS [36, 37]
		Family *Bacillaceae*	TAS [37, 38]
		Genus *Bacillus*	TAS [37–39]
		Species *Bacillus smithii*	TAS [2]
		Type strain: DSM 4216^T^	
	Gram stain	Positive^b^	TAS [2]
	Cell shape	Rod	IDA (Fig. 1), TAS [2]
	Motility	Motile	TAS [2]
	Sporulation	Terminal or sub terminal, oval or cylindrical endospores, non-swollen to slightly swollen sporangia	IDA (Fig. 1), TAS [2]
	Temperature range	25–65 °C	TAS [2]
	Optimum temperature	55 °C	IDA
	pH range; Optimum	5.5–6.8; 6.5	TAS [2], IDA
	Carbon source	D-glucose, D-xylose, L-xylose, L-arabinose, D-ribose, glycerol, D-adonitol, D-fructose, L-sorbose, D-galactose, L-rhamnose, inositol, D-mannitol, sucrose, D-trehalose, xylitol, Methyl-α-D-glucopyranoside, esculin, salicin, D-maltose, D-turanose, D-lyxose, D-tagatose, D-arabitol, K-gluconate, K-5-ketogluconate	IDA(API), TAS [2]
MIGS-6	Habitat	Type strain: cheese. Other strains: evaporated milk, canned food, compost, hot spring soil, sugar beet juice from extraction installations.	TAS [2, 9–11]
MIGS-6.3	Salinity	Not in 3 % NaCl (w/v)	TAS [2]
MIGS-22	Oxygen requirement	Facultative anaerobe	TAS [2]
MIGS-15	Biotic relationship	Free-living	TAS [2]
MIGS-14	Pathogenicity	Non-pathogen	TAS [12, 13]
MIGS-4	Geographic location	USA	TAS [2, 40]
MIGS-5	Sample collection	~1946	TAS [2, 40]
MIGS-4.1	Latitude	Unknown	
MIGS-4.2	Longitude	Unknown	
MIGS-4.4	Altitude	Unknown	

^a^Evidence codes – IDA: Inferred from Direct Assay; TAS: Traceable Author Statement (i.e., a direct report exists in the literature); NAS: Non-traceable Author Statement (i.e., not directly observed for the living, isolated sample, but based on a generally accepted property for the species, or anecdotal evidence). These evidence codes are from the Gene Ontology project

^b^As described in the species description by Nakamura et al.: “Young cells of both groups were Gram positive. With increasing age the cells became Gram variable and finally Gram negative. The KOH and aminopeptidase tests were negative, as is typical for Gram-positive organisms.”

**Fig. 2:**
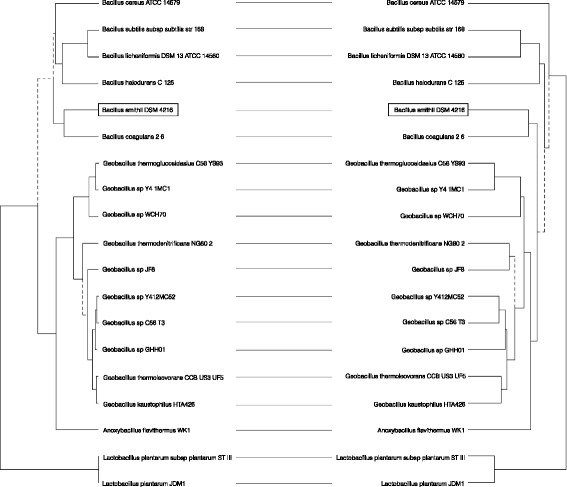
Phylogenetic tree based on 16S rRNA gene sequences (*left*) and protein domains (*right*). A comparison is included (horizontal lines) between the two trees, showing the position of *Bacillus smithii* DSM 4216^T^ relative to other *Bacillus* strains, as well as several industrially important Lactic Acid Bacterium strains. Only strains were used for which a complete genome sequence is available (as on 18 September 2014) in order to be able to perform the domain-based analysis. The 16S sequences were aligned using DECIPHER (R) [29] and the distance analysis was performed using a Jukes-Cantor correction. Phylogenetic analysis of all domains was performed by re-annotation of all proteins from selected genomes using InterProScan 5-RC7 and transformed into a absence-presence matrix. Distance was calculated using a standard Euclidean distance and clustering was performed by complete method using hclust. Tree comparison was performed by dendextend. Note that “unique” nodes between the 16S and domain-based tree are indicated with dashed lines (i.e. the order is the same but the subclustering is not). GenBank IDs of used whole genome sequences in order from top to bottom: AE016877.1, AL009126.3, CP000002.3, BA000004.3, CP012024.1, CP002472.1, CP002835.1, CP002293.1, CP001638.1, CP000557.1, CP006254.2, CP002442.1, CP002050.1, CP004008.1, CP003125.1, BA000043.1, CP000922.1, CP002222.1, CP001617.1

In order to compare the *B. smithii*DSM 4216^T^ genome to other fully sequenced *Bacillus* genomes, a phylogenetic tree was constructed based on 16S rRNA genes and the analysis of protein domains of *B. smithii*DSM 4216^T^ and other currently available *Bacillus* genomes (Fig. 2) [8]. These analyses indicated that *B. smithii* is most closely related to *B. coagulans*, which is also a facultatively thermophilic species [2].

The *B. smithii* type strain DSM 4216^T^ was isolated from cheese [1, 2], but other *B. smithii* strains have been isolated from compost [3, 9], hot spring soil [10], and a sugar beet factory [11]. It is a free-living organism that was shown to be non-cytotoxic [12]. In addition, the safety of the probiotic *B. smithii* TMBI 12 was recently reported in piglets studies [13]. Basic morphological and physiological features have been described by Nakamura et al.[2]. Genetic accessibility, a wide temperature and pH range and the ability to utilize a wide range of carbon sources in a relatively minimal medium make *B. smithii* an interesting new host for biotechnological applications [3, 4].

## Genome sequencing information

### Genome project history

The *B. smithii* type strain was selected based on the biotechnological relevance of the species as described above. The initial Illumina sequencing was performed in March 2012 and the genome was closed by PacBio sequencing in June 2013. The final, closed genome sequence consisting of 1 chromosome and 1 plasmid was deposited in GenBank (nr CP012024.1 and CP012025.1) and released for public access on 8 July 2015. A summary of the project information and its association with MIGS version 2.0 compliance [14] is shown in Table 2.

**Table 2:** Project information of the whole genome sequence of *B. smithii* DSM 4216^T^

MIGS ID	Property	Term
MIGS 31	Finishing quality	Finished
MIGS-28	Libraries used	Mate-pair (average 4,260 bp), paired-end (average 273 bp), PacBio (2,075 and 2,775 kbp)
MIGS 29	Sequencing platforms	Illumina and PacBio
MIGS 31.2	Fold coverage	Illumina paired-end: 187x, Illumina mate pair: 311x, PacBio: 56x
MIGS 30	Assemblers	CLCbio Genomics Workbench 5.0, SSPACE Premium 2.0, GapFiller 1.10
MIGS 32	Gene calling method	RAST and domain analysis
	Locus Tag	BSM4216
	Genbank ID	CP012024.1 (chromosome); CP012025.1 (plasmid)
	GenBank Date of Release	8 July 2015
	GOLD ID	NA
	BIOPROJECT	PRJNA258357
MIGS 13	Source Material Identifier	Biotechnological
	Project relevance	DSM 4216^T^

### Growth conditions and genomic DNA preparation

*B. smithii*DSM 4216^T^ was obtained from DSMZ. DNA was isolated from *B. smithii*DSM 4216^T^ cultures grown overnight at 55 °C in 100 mL LB2 and TVMY-glucose [3] in a 250 mL Erlenmeyer. 10 mL of the cultures was harvested by centrifugation for 15 min at 4 °C and 4816 × *g*, after which DNA was isolated using the Epicentre Master Pure Gram Positive DNA Purification kit according to the manufacturer’s protocol. DNA integrity was confirmed on a 1.0 % agarose gel and concentrations were measured using Qubit (Life Technologies), after which DNA integrity was re-evaluated by the sequencing company before sequencing.

### Genome sequencing and assembly

The genome of *B. smithii*DSM 4216^T^ was sequenced by BaseClear BV (NL) using Illumina HiSeq2000 mate-pair and paired-end sequencing for the initial sequencing and assembly, followed by PacBio sequencing to fully close the genome sequence. The average length of the paired-end samples was 273 bp and that of the mate-pair samples 4260 bp. The sequence reads were filtered and trimmed based on Phred quality scores, assembled into contigs using the “De Novo Assembly” option of the CLCbio Genomics Workbench version 5.0 and further assembled into scaffolds using SSPACE Premium version 2.0 [15]. This initial sequencing resulted in 6,185,516 reads, which were assembled into 214 contigs and 27 scaffolds. The coverage of the paired-end reads was 187x and that of the mate pair reads was 311x. For gap closure, sequencing was performed using a PacBio SMRT cell and quality was again assessed based on Phred scores. PacBio sequencing resulted in 90,013 reads with an average read length of 2075 kbp and a coverage of 56x. The contigs were assembled into super-scaffolds using alignment of the PacBio reads with BLASR [16], which was then used to determine the order of and distance between the contigs using a modified SSPACE Premium version 2.3 [15]. This resulted in 5 scaffolds, after which a second PacBio run was performed, which resulted in 114,294 reads with an average length of 2775 kbp. These results were analyzed in the same way as the first PacBio-round, after which gaps in the super-scaffolds were closed using GapFiller 1.10 [17], resulting in the final genome of 1 chromosome and 1 plasmid. Two small scaffolds (<450 bp) were found to be contaminants and removed from the data set.

Structural variations (SVs; small nucleotide polymorphism and small insertions and deletions) in the paired end and mate paired Illumina reads were compared to the PacBio scaffolds at the CMBI Nijmegen using an in-house developed tool RoVar [18]. Repeat masking of the reference sequence was done by (i) creating 30-bp fragments, (ii) aligning these fragments to the PacBio reference sequence by using BLAT [19] with a tile size of 6, and (iii) masking regions to which fragments align perfectly in multiple positions in the reference sequence. Illumina read alignment performed by BLAT with a tile size of 6 and alignment events were allowed provided that SVs were at least 4 bp from the end of a given read. SVs were used for further analysis provided that they were supported by at least 5 unique forward and 5 unique reverse reads and at most 1 % of the reads were allowed to suggest an alternative allele. A total of 14 SVs were corrected in the *B. smithii* 4216^T^ PacBio assembly.

### Genome annotation

The corrected PacBio assembly was subjected to RAST annotation [20] using default parameters. The following tools were used to predict gene functions (Table 4): Aragorn for tRNAs [21], RNAmmer for rRNA [22], and CRISPR-finder for CRISPR repeats and spacers [23]. The annotation was manually curated by running a BLAST of all genes and comparing starts and stops to the best hits. Via this method, also pseudogenes were manually identified.

As several pathways commonly found in bacilli were not identified by RAST in *B. smithii*, an analysis based on protein domains was performed on the *B. smithii*DSM 4216^T^ genome using InterProScan 5 (version 5RC7, 27th January 2014) (Koehorst & Van Dam, submitted for publication). This has been shown to be a powerful tool for identifying previously unknown protein functions, for example in determining microbial syntrophic interactions [8]. The domain-based annotation was compared to the manually curated RAST annotation, after which duplicates were removed and genes identified uniquely via the domain-analysis were added. In total 142 extra genes were annotated via this method, of which all except 4 were hypothetical proteins. For 209 genes, the protein domain annotation resulted in the addition of EC-numbers to the annotation that had not been assigned via RAST.

## Genome properties

**Fig. 3:**
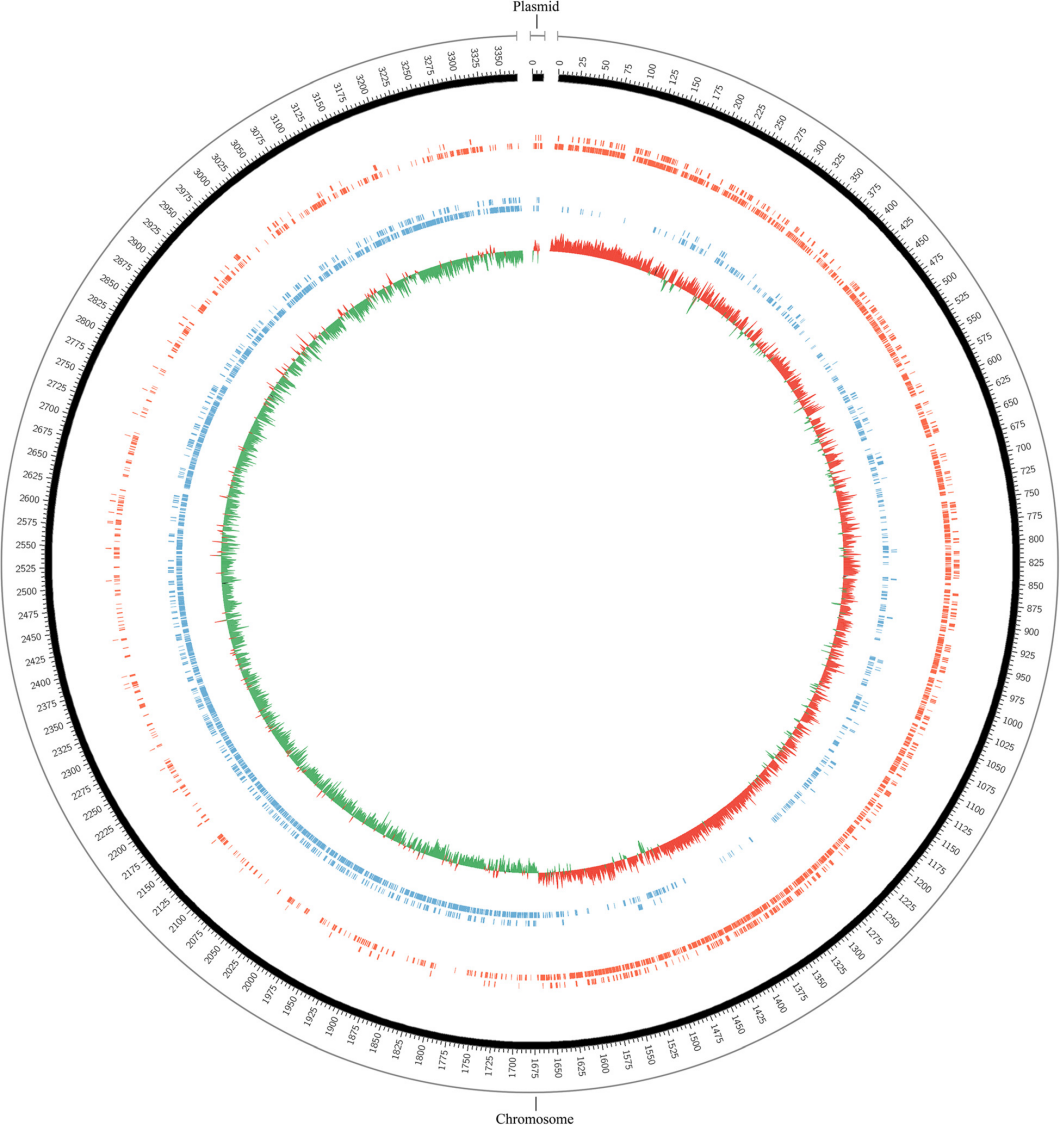
Chromosome and plasmid map of *B. smithii* DSM 4216^T^. The outer circle represents base pair numbers; red are genes on the forward strand and blue on the reverse; the inner circle represents GC skew in which red is a positive GC content and green a negative

The genome of *B. smithii*DSM 4216^T^ consists of a circular chromosome of 3,368,778 base pairs with a GC content of 40.8 % and a plasmid of 12,514 base pairs and a GC content of 35.9 % (Table 3). Figure 3 shows a map of the DSM 4216^T^ plasmid and chromosome. On the chromosome, a total of 3880 genes were identified, of which 3627 were annotated as protein-coding genes, of which 81 are assigned ‘putative’ or ‘probable’ functions, 1472 are hypotheticals or genes with unknown function (38.2 %) and the remaining had a defined function. Out of the total chromosomal genes, 126 genes are pseudogenes and 94 are tRNAs, 33 are rRNA genes, 122 are genes with signal sequences for secretion and 795 are genes with a transmembrane domain (Table 4). The rRNA genes are clustered in 11 operons, which is relatively many and is thought to be linked to the capacity to grow fast in different conditions [24]. Eight of these operons were found on the forward strand and 3 on the reverse strand. Six of the operons appear to be positioned approximately opposite of each other on the two strands, while the remaining five are located very closely to the origin and to each other on the forward strand. The plasmid DNA was predicted to contain 18 genes, of which 5 have a function assigned, 11 are hypotheticals and 2 are mobile element associated proteins. The COG-distribution of genes is shown in Table 5.

**Table 3:** Summary of the *B. smithii* DSM 4216^T^ genome: one chromosome and one plasmid

Label	Size (Mb)	Topology	INSDC identifier	RefSeq ID
Chromosome	3,368,778	Circular	CP012024.1	NZ_CP012024.1
Plasmid	12,514	Circular	CP012025.1	NZ_CP012025.1

**Table 4:** Genome statistics of *B. smithii* DSM 4216^T^

Attribute	Value	% of total
Genome size (bp)	3,381,292	100.0
DNA coding (bp)	2,799,365	82.8
DNA G + C (bp)	1,378,026	40.8
DNA scaffolds	2	
Total genes	3,880	100.0
Protein coding genes	3,627^a^	93.5
RNA genes	127	3.3
Pseudo genes	126	3.2
Genes in internal clusters	ND	
Genes with function prediction	2,063	53.1
Genes assigned to COGs	2,619	67.4
Genes with Pfam domains	2,596	66.8
Genes with signal peptides	122	3.1
Genes with transmembrane helices	795	20.5
CRISPR repeats	69	

^a^This is excluding 126 pseudogenes

**Table 5:** Number of genes associated with general COG functional categories

Code	Value	% age	Description
J	162	4.46	Translation, ribosomal structure and biogenesis
A	0	0.00	RNA processing and modification
K	179	4.92	Transcription
L	160	4.40	Replication, recombination and repair
B	1	0.03	Chromatin structure and dynamics
D	28	0.77	Cell cycle control, Cell division, chromosome partitioning
V	31	0.85	Defense mechanisms
T	125	3.44	Signal transduction mechanisms
M	132	3.63	Cell wall/membrane biogenesis
N	64	1.76	Cell motility
U	42	1.16	Intracellular trafficking and secretion
O	92	2.53	Posttranslational modification, protein turnover, chaperones
C	156	4.29	Energy production and conversion
G	174	4.79	Carbohydrate transport and metabolism
E	291	8.01	Amino acid transport and metabolism
F	74	2.04	Nucleotide transport and metabolism
H	107	2.94	Coenzyme transport and metabolism
I	94	2.59	Lipid transport and metabolism
P	154	4.24	Inorganic ion transport and metabolism
Q	70	1.93	Secondary metabolites biosynthesis, transport and catabolism
R	382	10.51	General function prediction only
S	236	6.49	Function unknown
-	1,321	36.34	Not in COGs

## Insights from the genome sequence

As the number of available genome sequences from thermophilic bacilli is still rather limited and *B. smithii* also grows at mesophilic temperatures, we compared its genome properties to those of thermophilic bacilli as well as to those of several commonly studied mesophilic bacilli (Table 6). Compared to its close relative *B. coagulans*, *B. smithii* has a slightly larger genome with a lower GC content. Compared to most mesophilic bacilli, its genome is smaller and it has a higher GC content than *B. cereus* but lower than *B. halodurans* and *B. subtilis*. As will be discussed in the next section, the genome content differs from its close relatives in several ways.

**Table 6:** Comparison of several published complete genome sequences of the genus *Bacillus*

Species/strain	Genome size (bp)	GC %^a^	ORFs^b^	Plasmid number	Growth^c^	Ref
*B. smithii* DSM 4216^T^	3,368,778	40.8	3,635	1	TT	This study
*B. coagulans* DSM1^T^*	3,018,045	47.2	3,437	0	TT	[41]
*B. coagulans* 36D1	3,552,226	46.5	3,306	0	TT	[42]
*B. coagulans* 2-6	3,073,079	47.3	2,985	1	TT	[43]
*A. flavithermus* WK1	2,846,746	41.8	2,863	0	TT	[44]
*B. licheniformis* 10-1	4,317,010	45.9	4,650	0	TT	[45, 46]
*B. licheniformis* DSM13^T^	4,222,748	46.2	4,286	0	TT	[47]
*B. cereus* ATCC 14579	5,426,909	35.3	5,366	1	MP	[48]
*B. halodurans* C-125	4,202,353	43.7	4,066	0	MP	[49]
*B. subtilis* 168^T^	4,214,810	43.5	4,100	0	MP	[50]
*G. thermoglucosidans* TNO-09.020*	3,75 Mb	43.9	4,300	0	TP	[51]
*G. thermodenitrificans* NG80-2	3,550,319	48.9	3,499	1	TP	[52]
*G. kaustophilus* HTA426	3,544,776	52.0	3,498	1	TP	[53, 54]
*G. thermoleovorans* CCB_US3_UF5	3,596,620	52.3	3,887	0	TP	[55]

Currently available thermophilic *Bacillus* genomes are shown, as well as a selection of genomes of mesophilic model organisms

*Sequence not fully closed

^a^GC% of chromosome and plasmid combined as weighted average

^b^Open Reading Frames as a total on chromosome and plasmid(s)

^c^MP: mesophile, TP: thermophile, TT: thermotolerant (grows at mesophilic as well as thermophilic temperatures)

### Central carbon metabolism and main product pathways

**Fig. 4:**
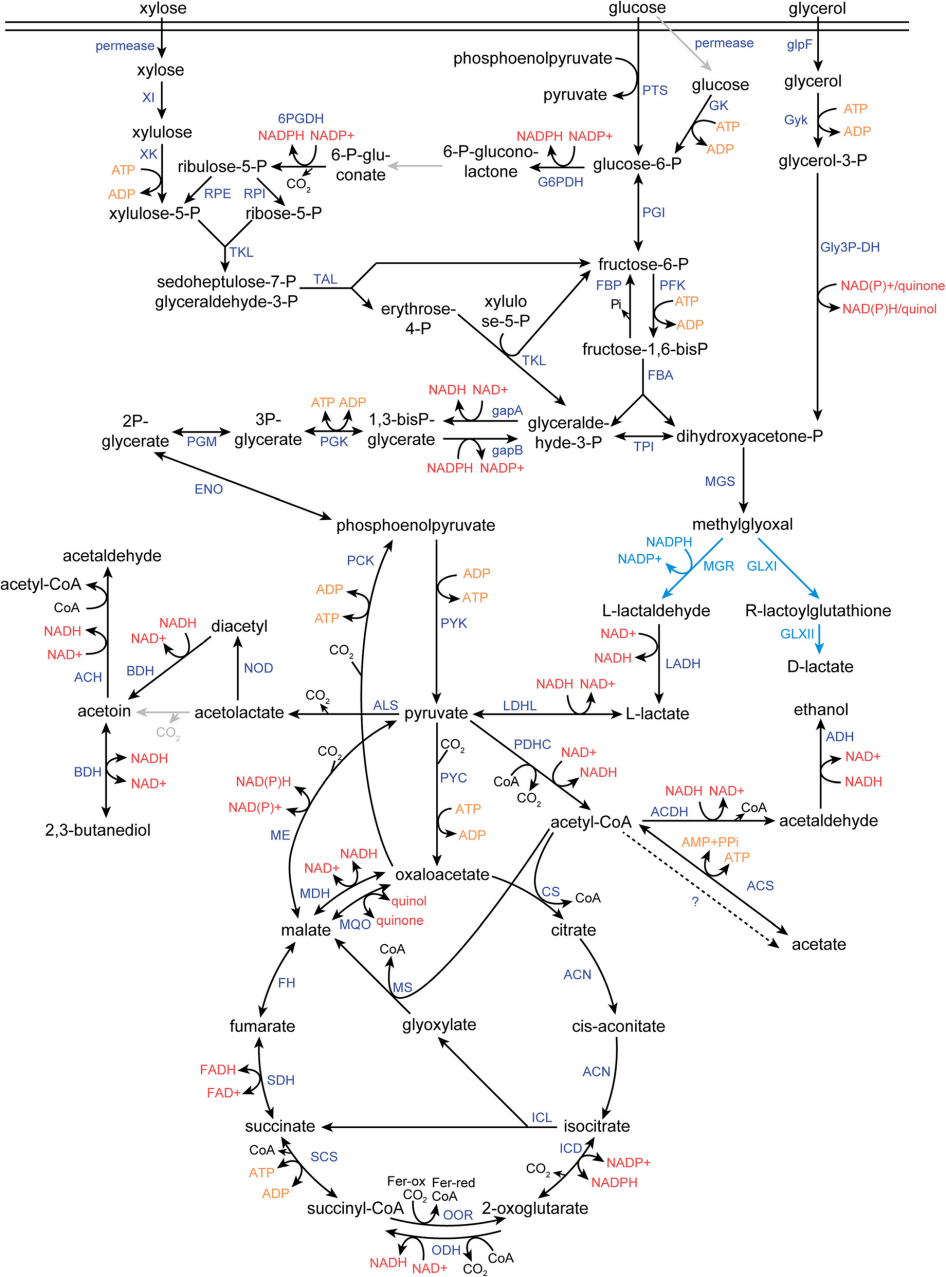
Reconstruction of central carbon metabolism of *B. smithii* DSM 4216^T^. Blue lines indicate pathways of which the EC-number was identified only via domainome analysis; grey lines indicate pathways unidentified by both RAST annotation and domainome analysis. Abbreviations: XI: xylose isomerase; XK: xylulokinase; PTS: phosphotransferase system; GK: glucokinase; glpF: glycerol facilitator; glyK: glycerol kinase; Gly3P-DH: glycerol-3-phosphate dehydrogenase; PGI: glucose-6-phosphate isomerase; G6PDH: glucose-6-phosphate dehydrogenase; 6PGDH: 6-phosphogluconate dehydrogenase; RPI: phosphopentose isomerase; RPE: phosphopentose epimerase; TKL: transketolase; TAL: transaldolase; FBP: fructose bisphosphatase; PFK: phosphofructokinase; FBA: fructose bis-phosphate aldolase; TPI: triosephosphate isomerase; GAP: glyceraldehyde 3-phosphate dehydrogenase; PGK: phosphoglycerate kinase; PGM: phosphoglycerate mutase; ENO: enolase; PCK: phosphoenol pyruvate carboxykinase; PPC: phosphoenol pyruvate carboxylase; PYK: pyruvate kinase; PYC: pyruvate carboxylase; PDHC: pyruvate dehydrogenase complex; ME: malic enzyme; MDH: malate dehydrogenase; MQO: malate:quinone oxidoreductase; CS: citrate synthase; ACN: aconitase; ICL: isocitrate lyase; MS: malate synthase; ICD: isocitrate dehydrogenase; OOR: 2-oxoglutarate reductase; ODH: 2-oxoglutratae dehydrogenase; SCS: succinyl-CoA synthetase; SDH: succinate dehydrogenase; FH: fumarate hydratase; ALS: acetolactate synthase; NOD: non-enzymatic oxidative decarboxylation; BDH: butanediol dehydrogenase; ACH: acetoin dehydrogenase; LDHL: L-lactate dehydrogenase; ACDH: acetyl-CoA dehydrogenase; ADH: alcohol dehydrogenase; ACS: acetyl-CoA synthetase; MGS: methylglyoxal synthase; MGR: methylglyoxal reductase; GLXI: glyoxalase I; GLXII: glyoxalase II; LADH: lactaldehyde dehydrogenase

To be able to use *B. smithii* as a host for biotechnological purposes, it is important to understand its metabolic pathways. In the *B. smithii*DSM 4216^T^ genome, all genes involved in glycolysis, gluconeogenesis, pentose phosphate pathway, TCA-cycle and glyoxylate shunt could be identified, but not the complete sets of genes for the phosphoketolase and Entner-Doudoroff pathways. Uptake systems for all sugars shown to support growth in the API-test were annotated by the RAST annotation. The organization of the xylose catabolic operon is similar to that found in *B. coagulans* XZL4 [25]. A reconstruction of the central carbon metabolism of *B. smithii*DSM 4216^T^ is shown in Fig. 4. An L-lactate dehydrogenase gene was annotated, which is in accordance with L-lactate being the major fermentation product of *B. smithii* [3, 4]. After RAST annotation, the methylglyoxal pathway was identified only towards D-lactate, but an in-depth analysis of protein domains also revealed the presence of all genes necessary for L-lactate production via methylglyoxal. Based on 16S rRNA gene and complete protein domain analysis (Fig. 2), the closest relative of *B. smithii* is *B. coagulans*. However, when reconstructing the metabolic network of *B. smithii*, several remarkable differences between *B. smithii* and *B. coagulans* as well as other bacilli were observed. The most striking difference with bacilli in general is the absence of the genes coding for phosphotransacetylase and acetate kinase, which form the standard acetate production pathway in bacteria. This was confirmed by the domain-based analysis. Moreover, we also could not identify these two genes in the genome sequence of *B. smithii* strain 7_3_47FAA, which is available from a metagenome database. The fact that *B. smithii* produces significant amounts of acetate from glucose [3, 4] indicates that an alternative pathway is involved, which is currently being investigated. Furthermore, candidate genes for pyruvate-formate lyase, pyruvate decarboxylase and pyruvate oxidoreductase could not be found in the genomes of both DSM 4216^T^ and 7_3_47FAA via either RAST or domain-based analysis. Therefore, *pdhc*-encoded pyruvate dehydrogenase complex is most likely the only enzyme responsible for the conversion of pyruvate to acetyl-CoA. This is confirmed by a *pdhA*-knockout strain of *B. smithii* strain ET 138, which is unable to grow without acetate supplementation and did not produce any acetate [4].

Another difference with *B. coagulans* is the lack of a catabolic *alsSD*-operon in *B. smithii*, coding for the enzymes acetolactate synthase and acetolactate decarboxylase. This is in accordance with the absence of 2,3-butanediol production [3, 4]. The anabolic acetolactate synthase small and large subunit genes *ilvBH* (also called α-acetohydroxyacid synthase) are present. These genes are mainly involved in the isoleucine and valine biosynthetic pathways [26]. On the other hand, both an S- and an S/R-acetoin specific 2,3-butanediol dehydrogenase gene were identified in the genome. Although several alcohol dehydrogenases were found in the genome, no bifunctional acetaldehyde dehydrogenase-alcohol dehydrogenase *adhE* could be found, which is in accordance with the absence of alcohol production in the majority of *B. smithii* fermentations [3].

### Amino acid and vitamin biosynthesis pathways

Microorganisms used for biotechnological purposes should have minimal nutrient requirements, as the addition of yeast extract, vitamins or amino acids is costly. The organisms should therefore preferably contain the pathways to synthesize vitamins, amino acids, purines and pyrimidines. In *B. smithii*DSM 4216^T^, all amino acid biosynthetic pathways could be identified. Pathways for *de novo* synthesis and salvage pathways of pyrimidines and purines were also identified. Complete vitamin biosynthesis pathways were identified for cobalamin, riboflavin, tetrahydrofolate, panthothenate, *p*-aminobenzoic acid, nicotinic acid and pyridoxal, but not for thiamine, ascorbate, pyridoxamine and D-biotin.

### Host-defense systems

Robustness against infection is crucial for industrial microorganisms. Host-defense systems can confer such robustness, but might also hinder genetic accessibility of the organism. In the genome of *B. smithii*DSM 4216^T^, several host-defense systems are annotated: a type II-s restriction endonuclease, a 5-methylcytosine-specific restriction related enzyme, a type I restriction-modification system and a CRISPR-Cas Type I-B system. The CRISPR-Cas genes show the typical type I-B gene arrangement [27], but seem to be partly duplicated around the CRISPR locus with a second locus containing cas6, cas8a/cst1, cas7 (originally annotated as ‘CRISPR-associated negative autoregulator') and cas5 after the CRISPR repeats. The CRISPR-finder tool [23] was used to identify CRISPR repeats and spacers in the area around the Cas-genes (bp 2,772,457-2,799,872). Three CRISPR-loci were identified (CRISPR 1, 3 and 4) as well as one questionable locus (CRISPR 2). Using CRISPRTarget [28], some of the spacers were found to have hits with potential protospacer target sequences against *Bacillus**sp.* and *B. subtilis* plasmid DNA, and against *Streptococcus thermophilus*, *Lactococcus*, *Enterococcus* and *Campylobacter* phage DNA.

## Conclusions

This report describes the complete genome sequence of *Bacillus smithii* type strain DSM 4216^T^. The species has biotechnological potential due to its efficient conversion of both C_5_ and C_6_ sugars at 55 °C to lactic acid, combined with its genetic accessibility. Its central carbon metabolism is different from its close relative *B. coagulans* as it lacks the *alsSD* operon, as well as the *pta-ack* acetate production pathway and the *pfl* gene.

## Abbreviations

DSMZ: Deutsche Sammlung von Mikroorganismen und Zellkulturen
SVs: Structural variations
